# Knowledge, perceptions, and exposure to bats in communities living around bat roosts in Bundibugyo district, Uganda: implications for viral haemorrhagic fever prevention and control

**DOI:** 10.1186/s12879-024-09162-x

**Published:** 2024-03-14

**Authors:** Lesley Rose Ninsiima, Luke Nyakarahuka, Steven Kisaka, Collins GK. Atuheire, Lawrence Mugisha, Terence Odoch, Javier Sánchez Romano, Jörn Klein, Siobhan M. Mor, Clovice Kankya

**Affiliations:** 1 Department of Biosecurity, Ecosystems and Veterinary Public Health, Makerere University, Kampala, Uganda; 2 Department of Livestock Industrial Resources, Makerere University, Kampala, Uganda; 3 Department of Medical Biology, UiT The Artic University of Norway, Tromsø, Norway; 4 University of South Eastern Norway, Porsgrunn, Norway; 5 Institute of Infection, Veterinary and Ecological Sciences, University of Liverpool, Liverpool, UK; 6 International Livestock Research Institute, Addis Ababa, Ethiopia

**Keywords:** Knowledge, Perceptions, Bats, Bat-exposure, Bat roosts, Uganda

## Abstract

**Background:**

Bats are a reservoir for many viruses causing haemorrhagic fevers. Proximity to bats is a risk factor for virus spillover to animals and humans. We conducted this study to assess knowledge, perceptions, and exposure to bats in communities living near bat roosts in Bundibugyo District, Uganda.

**Methods:**

A cross-sectional study using mixed methods with both quantitative and qualitative data was conducted between September and December 2022. Participants for the quantitative data (survey) (*n* = 384) resided near bat caves and/or roost sites and were selected using multistage random sampling. The survey investigated participants’ prior exposure to bats, as well as knowledge and perceptions of bat exposure. Logistic regression was used to determine factors associated with bat exposure. Participants for the qualitative data (focus group discussions) (*n* = 10, 6–8 participants each) were purposely selected based on engagement in guano mining, hunting, and farming activities. Perceived risk associated with bat-related activities were identified and ranked in the focus group discussions using participatory epidemiology tools.

**Results:**

In total, (214/384, 55.7%) had a history of bat exposure and (208/384, 54.2%) had poor knowledge of risk factors associated with bat exposure. Increased exposure to bats was associated with being male (OR = 1.6; 95% CI: 1.0, 2.4 *p*-value = 0.038), staying in urban areas (OR = 1.9; *p*-value = 0.010), hunting (OR = 10.9; *p*-value = 0.024), and positive perception to bat guano being safe as fertiliser (OR = 2.5; *p*-value = 0.045). During the proportional piling process, a total of 7 risk factors were identified by 10 groups with hunting during an outbreak and consumption of bats being the most frequently identified. Overall, there was a strong statistical agreement in the ranking across the 10 focus groups (W = 0.52; *p* < 0.01; *n* = 10). Based on the provided data, the adjusted odds ratio of 0.7 for the good measures (*p*-value = 0.112), suggests a potential protective effect on the risk of bat exposure.

**Conclusion:**

Communities living around bat roosts frequently come into contact with bats, yet there is inadequate awareness regarding the behaviors that can lead to the transmission of bat- borne diseases to humans. It is essential to undertake educational initiatives and preventive measures to minimise the risks of bat-related infections. The need for targeted health communication and education efforts to address these knowledge gaps and promote an accurate understanding of bats and disease transmission. Understanding of diseases associated with bats will minimize bat-related health risks especially in communities engaged in wildlife hunting.

**Supplementary Information:**

The online version contains supplementary material available at 10.1186/s12879-024-09162-x.

## Introduction

Bats (order: Chiroptera) are the second largest group of mammalian species after rodents in terms of number, with over 1400 species recorded to date [1]. They are a key component of cave ecosystems, where they are part of a diverse vertebrate community [2]. Additionally, their assemblages are useful indicators of habitat quality and disturbance [3]. Ecologically, bats play roles in pollination, seed dispersal, and pest control [4]. However, bats are also recognized as important reservoirs of viruses that are harmful to human and animal health, posing potentially serious public health risks to adjacent communities and susceptible animal populations [5]. Bats have been documented as natural hosts of many diverse viruses such as lyssaviruses, paramyxoviruses, and filoviruses [6]. Although some studies have demonstrated that exposure to bats among communities staying near bat roosts was common, bat’s role in disease transmission to these communities is largely neglected [1, 7, 8].

Several diseases are thought to have a bat reservoir, including Marburg virus disease (MVD), Ebola virus disease (EVD), Nipah virus disease, rabies, severe acute respiratory syndrome (SARS) and Middle East respiratory syndrome (MERS) [7, 9]. With the exception of rabies, outbreaks of these diseases are usually associated with human-to-human transmission following spillover from a bat or intermediate animal host [10, 11]. The EVD outbreak that affected several countries in West Africa between 2014–2016 and resulted in 28,616 cases and 11,310 deaths is thought to have originated following direct contact with bats [12]. In an exploratory study carried out among 135 humans in southern Cameroon, direct human–bat contacts were found to be substantial: 40% of respondents reported consuming bats, 28% hunted them, 17% reported being previously bitten by bats, and 22% reported that children catch them [1]. The health risks to communities posed by bats are exacerbated by the attitudes and practices of residents. Many bat species and their roosts occur inside human habitations and outside legally protected areas, where they are highly prone to direct persecution and roost destruction [13]. Poor attitudes of people towards bats coupled with hunting of bats for food and medicinal purposes potentially exposes communities to infectious agents as well as threaten the long-term viability of local bat populations [14]. Understanding the level of knowledge and attitude of community towards bats is thus important to design effective community awareness campaigns aimed at preventing spillover [15].

Throughout generations, a multitude of beliefs, values, myths, and historical narratives have been woven into the symbolic realm of bats. These elements exert a significant influence on how people perceive and value bats [2]. The hunting and eating of bushmeat by humans, however, carries a substantial risk for cross-species transmission of disease [1, 5] especially where bats are involved. Negative perceptions largely overshadow any understanding of the beneficial ecological roles of bats and result in negative attitudes, reduced empathies, and direct persecution of bats in many regions [16]. Thus, it is pertinent that the perceptions, knowledge, and several other factors surrounding human-bat interactions are investigated to better understand how the interplay of these factors may affect both bats and humans sharing the same ecosystem.

Uganda has experienced multiple outbreaks of MVD and EVD over recent decades. In 2007 for example, after reports of a mysterious illness in Bundibugyo District, a novel ebolavirus species, subsequently named Bundibugyo ebolavirus (BEBOV), was identified [17]. The outbreak of BEBOV was associated with 149 cases and 37 deaths. Most recently, an outbreak of Sudan ebolavirus (SEBOV) originating in Mubende district spread to several other districts between September 2022 and January 2023, infecting 160 people and resulting in 77 deaths before it was brought under control [18]. There are several areas in Uganda where there are high densities of bats, some of which have experienced outbreaks of hemorrhagic fevers [17]. This includes Bundibugyo District, which has several caves and different roosting sites that provide suitable roosting habitat for bats. Bundibugyo district is continuously occupied with multiple species of bats including several hundred Angolan soft-furred fruit bats (*Lissonycteris angolensis ruwenzorii* Bocage, 1898) and a population of Sundevall’s leaf-nosed bats (*Hipposideros caffer* Sundevall, 1846) [19]. Some of the common fruit bat species found in the farmlands of Bundibugyo are Wahlberg’s Epauletted Fruit Bat (*Epomophorus wahlbergi*) and Egyptian Rousette (*Rousettus aegyptiacus*), while the insectivorous bats are Heart-nosed Bat (*Cardioderma cor*), Egyptian Slit-Faced Bat (*Nycteris thebaica*), Striped Leaf-nosed Bat (*Macronycteris vittata*) and Green House Bat (*Scotophilus viridis*). All of these bat species roost in natural and man-made structures [13, 19]. We undertook a study to better understand the knowledge, perceptions, and exposure to bats in communities living around bat roosts in Bundibugyo District, Uganda.

## Materials and methods

### Study design

A cross-sectional study using mixed methods to collect both quantitative and qualitative data was conducted in Bundibugyo District between September and December 2022. For quantitative data, a survey was done and for qualitative data, we used focus group discussions.

### Study area

**Fig. 1:**
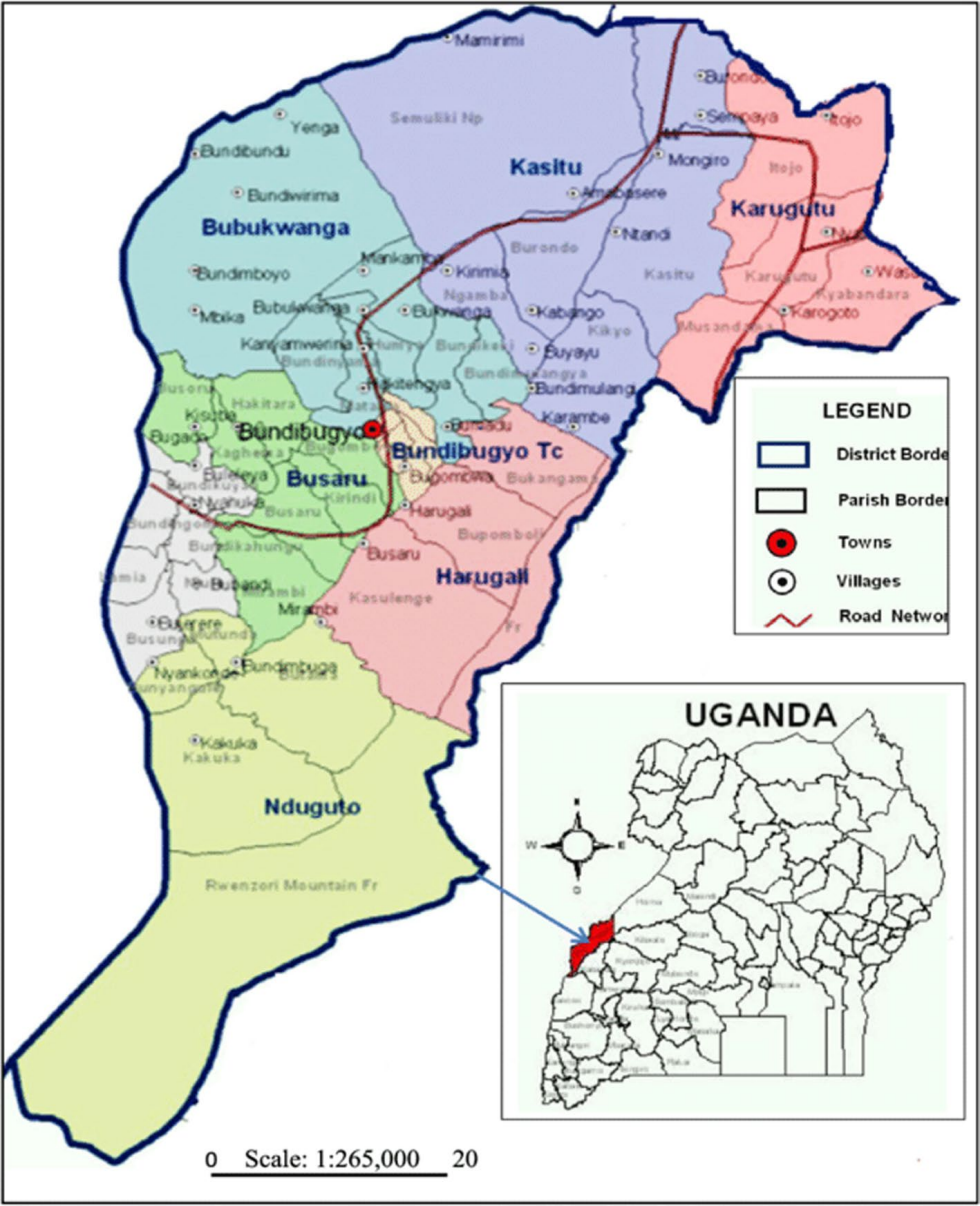
A map of Uganda showing Bundibugyo district (Adopted)

Bundibugyo District is one of the districts of the Rwenzori region, in western Uganda as shown in Fig. 1 below. The district borders the Democratic Republic of the Congo (DRC) and lies in the west of the Rwenzori mountains. In the past, the western Albertine Rift, which encompasses the Rwenzori region, has been referred to as a hotspot for zoonotic disease outbreaks because of its geographical makeup and its cross-border trade with the Democratic Republic of the Congo [20]. Agriculture is a significant contributor to the district economy with the main cash crops (Cocoa, Vanilla and Palm oil), vegetables and fruits [16]. The town of Bundibugyo is located approximately 378 kms (235miles), by road, west of Kampala, the largest city and national capital of Uganda as shown in the figure below.

### Study population

The target population included all residence in the three selected sub counties aged 18 years and above. Residency requirement was defined as residing in a household that is located near a bat roosting site. For Focus Group Discussion, participants were eligible if they were aged 18 years or older and engaged in at least one of these activities (hunting, farming, guano mining within the last 5 years.

### Inclusion and exclusion criteria

Inclusion criteria included willingness to participate in the study, age 18 years and above, able to understand English, Lugwisi and/or Lukonjo and ability to understand the study objectives. People who declined to participate or who had a history of mental disorder were excluded. For those who declined to participate, selection of the next nearest house was done (i.e., sampling with replacement).

### Sample size and sampling

#### Quantitative

Since no studies have previously explored knowledge and perceptions of human-bat interaction, we used *P* = 0.5 to denote the expected level of knowledge and perceptions, thus giving a sample size of 384, according to the Leslie Kish’s formula n = Zα^2* P (1-P) / d^2 [20], assuming 95% confidence and 5% precision. Multi-stage sampling was used to select participants for this study with sub counties selected based on proximity to bat caves and/or mass bat roosting sites and certain community characteristics known to promote bat associated activities. In the first stage, three sub-counties (Ntandi, Burondo, and Harugale) were purposively selected in consultation with the district veterinary officer who confirmed these were the only sub-counties in Bundibugyo district where caves were present. A map of coordinates of all the households near the bat roosting sites was available in each sub-county. Sampling was initiated by generating a random coordinate within each of the three sub counties with reference to the nearest subcounty to the district headquarters (Burondo subcounty) and selecting the house nearest to this coordinate. Sampling continued with the next nearest house and so on until the sample size was reached (*n* = 128 households per sub-county). From each selected household, one eligible participant was selected at random.

#### Qualitative

Participants for the focus group discussion were purposively selected from rural and urban localities of the three sub-counties targeted for the survey (i.e., Ntandi, Burondo, and Harugale). Village leaders and other local contacts aided in locating individuals known to engage in guano mining, hunting, and farming. Ten focus groups were held, each comprising 6–8 participants. The sample size provided enough breadth to reach saturation in terms of identifying perceived risk factors.

### Data collection

For quantitative data, we used a semi-structured questionnaire which was designed in English and translated into Lugwisi and Lukonjo, which are the two most spoken ethnic languages in Bundibugyo district. The questionnaire explored participants' prior exposure to bats, including self-reported ‘handling of bats’, being bitten by bats and consumption of bats, as well as data on risk behaviors and demographic characteristics to allow comparison between respondents who did and did not report exposure to bats. Results from the pre-test were not included in this study. The questionnaire was delivered by face-to-face interview of the participants in their households by trained research assistants. Data was collected from a personal digital assistant equipped with Global positioning system (GPS) using Kobocollect software.

For qualitative data, we used Focus Group Discussion which were conducted from 10 villages which were selected purposively within Bundibugyo district comprising of 6–8 respondents. The villages were selected based on their cultural interaction with bats such as hunting and eating the bats. Risk perception was assessed during the focus group discussion using participatory epidemiology tools. Groups were firstly invited to list 4 or 5 activities that they identified as risk factors for exposure to bat-borne diseases. The perceived importance of each of the activities in terms of exposure risk was then assessed using proportional piling. Groups were asked to distribute 100 beans across the 5 or 6 named activities according to how they perceived the risk of disease transmission from bats. To verify that participants understood the activity correctly, once all beans had been allocated, the group was asked to confirm which exposure ranked high and low in terms of transmission risk. Any discrepancies were resolved with discussion and re-allocation of beans where needed.

### Data analysis

#### Quantitative

After data collection was completed, answers to open-ended questions were grouped and coded for analysis. The knowledge, perception and exposure to bats assessment was executed using a scoring system. Knowledge and perception questions that were good/positive were scored one (1) while those that were poor/negative were scored zero (0) respectively. All questions were given equal weight, and missing and not applicable responses were excluded from the analysis, whereas “Don’t know” responses were scored zero (0). The knowledge and perception scores for each study participant were used to compute the total scores out of a total score of 6 and 12 respectively. For knowledge, the total score for knowledge was obtained by summating the raw score of each item and ranged from 0 to 6, with an overall greater score indicating accurate good knowledge. A cut-off level of more than or equal to 3 was set for categorizing the good knowledge. For perception, the total score was calculated by summating the raw scores of the 12 questions ranging from 12 to 36, with an overall higher score indicating more positive perception. A cut-off level of more than or equal to 23 was set for a more positive perception. The validity of the knowledge and perception questions was confirmed by an adequate Cronbach’s alpha internal consistency measured at 0.89 during the piloting phase [21]. To assess potential bat exposures, participants were asked questions about if they were bitten or scratched by a bat, bats in the household (dead or alive) and consumption of bats. These questions were coded to define exposure with (0) for no exposure and (1) for exposure. To assess knowledge and perception and bat exposure risk, variables of interest were compared between exposed and non-exposed persons using odds ratios (OR) with 95% confidence intervals (CI). *P*-values were calculated with the chi square test as appropriate. Data were summarized using descriptive statistics, and comparisons were made using Chi-square test. Comparisons yielding a *p*-value of < 0.1 were included in subsequent multivariate analysis. Bivariable and multivariable logistic regression analyses were used to explore factors for knowledge and perception independently (variable = knowledge and perception towards bats) associated with bat exposure. Variables related to the outcome at *p*-values = 0.05 were included in the model. Crude and adjusted odds ratios (OR) with 95% confidence intervals (CI) were calculated. Associations were statistically significant at *p*-values less than 0.05 (α = 0.05). All statistical tests were performed using STATA software version 15.0.

### Qualitative

Information collected based on the average number of beans from each group was ranked with high, medium, and low risk factors to bat borne diseases. Each group was tasked to identify 5–6 risk factors and rank them. The overall rank for each activity was summarized using mean and ranked from the largest to the lowest. Level of agreement between focus groups was assessed using Kendall’s coefficient of concordance (W) [22].

## Results

### Study participants

Table 1 shows the characteristics of the survey participants. The majority of respondents were adult men (*n* = 212, 55.2%) and most were from rural areas (*n* = 255, 66.4%). Most of the respondents were farmers (*n* = 231, 60.2%), and hunters *(n* = 20, 5.2%) and the rest of the respondents were involved in a variety of other occupations like business and civil service (*n* = 133, 34.6%). In terms of educational attainment, around a half had completed primary school (*n* = 197, 51.3%) while 26.5% had completed secondary or tertiary education; the remaining participants had no formal education (*n* = 85, 22.1%). Most of the respondents (*n* = 369, 96%) had lived in the area for more than a year. Around one quarter (24.5%) claimed to earn less than 15,001 Ugandan shillings per month (4 US dollars) which is below the international poverty line, while the majority of respondents’ (*n* = 161, 42%) family income was between 15,000 and 40,000 Ugandan shillings per month.

**Table 1:** Socio-demographic characteristics of participants in a survey of knowledge, perceptions, and exposure to bats in Bundibugyo district, Uganda

Sociodemographic variables	Frequency n (%)
**Sex**	
Female	172(44.8)
Male	212(55.2)
**Area setting**	
Rural	255(66.4)
Urban	129(33.6)
**Occupation**	
Farmer	231(60.2)
Hunter	20(5.2)
Other	133(34.6)
**Duration (more than one year)**	
No	15(4.0)
Yes	369(96.0)
**Marital status**	
Cohabiting/Married	311(81.0)
Divorced/separated/Widowed	31(8.0)
Single	42(11.0)
**Education**	
Tertiary	22(5.7)
Secondary	80(20.8)
Primary	197(51.4)
None	85(22.1)
**Family income (Ugx per month)**^**a**^	
< 15,001	94(24.5)
15,001 – 40,000	161(42.0)
40,001 – 70,000	71(18.5)
> 70,000	58(15.0)

^a^*1USD* 3600Ugx (current exchange rate)

Focus Group Discussion were conducted with both women and men in Bundibugyo district. The number of participants at each meeting ranged from 6 to 8 participants. Most participants who attended from each group were men as the activities that were in the inclusion criteria were mostly done by the men.

### Frequency of exposure to bats

More than half of the survey respondents reported that they had a history of exposure to bats (55.7%, *N* = 214). Among the exposed individuals, 56.5% (121/214) reported being bitten or scratched by bats, while 32.3% (69/214) reported consuming bats. Fewer participants (24/214, 11.2%) reported having bats in their households (either dead or alive).

### Knowledge aspects towards bat exposure among persons living near bat roosting sites in Bundibugyo district

Table 2 shows that over half of the participants reported poor knowledge (55.21%, 212/384). Around while (71.35%, 274/384) of the respondents believe that people cannot get diseases from bats. Most identified bat roosts were caves (196/383, 51.18%) and farms (171/383, 44.65%) while only a small percentage identified buildings (16/383, 4.18%) as bat roost. The majority of the respondents mentioned that the reasons for visiting bat roosting sites are to collect water (177/384, 46.21%) and collect guano (90/384, 23.5%). The most used health communication channels are radios (107/384, 53.65%) and village health teams (206/384, 27.86%). Among the measures cited to prevent bat-borne diseases, regular hand washing (39.21%, 149/384) and avoiding consumption of wildlife (bats) (24.74%, 94/384).

**Table 2:** The knowledge aspects of survey participants towards bat exposure among persons living near bat roosting sites in Bundibugyo district

Knowledge	Variables	Frequency(*n* = 384)	%
**Can people get diseases from bats**	Yes^a^	110	28.65
	No^b^	274	71.35
**Measures put in place to keep bats borne diseases**	Nothing^b^	21	5.53
	Avoid eating wildlife (bats)^a^	94	24.74
	Drinking boiled water^a^	37	9.74
	Washing hands regularly^a^	149	39.21
	Others^a^	79	20.79
**Bat roosting sites**	Caves^a^	196	51.18
	Buildings^a^	16	4.18
	Farms^a^	171	44.65
**Why people go where bats roost**	To collect guano^a^	90	23.5
	To collect water^a^	177	46.21
	To hunt for bats^a^	14	3.66
	For recreation^a^	49	12.79
	For religious activities^a^	53	13.84
**Health communication channels**	Village Health teams^a^	107	27.86
	Radios^a^	206	53.65
	Others^a^	57	14.84
	None^b^	14	3.65
**Knowledge overall**	Poor	212	55.21
	Good	172	44.79

^a^goog knowledge

^b^Poor knowledge

### Perceptions towards bat exposure among persons living near bat roosting sites in Bundibugyo district

Table 3 shows that more than half of the respondents (261/384, 67.97%) believe that touching a bat can lead to disease transmission and majority (281/384, 73.18%) believe that people can get diseases from bats by sharing drinking water with them. More than half (239/384, 62.24%) of the respondents were unsure of the economic importance to bats, and (42.71%, 164/384) expressed confidence in the safety of using bat guano as fertilizer. Majority of the participants (335/384, 87.24%) believed it's not okay to touch a dead bat and majority (355/384, 92.45%) would not cook a dead bat as sauce. A small percentage (22/384, 5.73%) reported bringing home and cooking dead bats. Respondents reported not allowing their children to touch bats (322/384, 83.85%). Among the participants, (63.19%, 242/384) reported a negative perception towards bats.

**Table 3:** The perception of survey participants towards bat exposure among persons living near bat roosting sites in Bundibugyo district

Perception	Variables	Frequency(*n* = 384)	%
**Bat guano is safe to use as fertilizer**	Don’t know^b^	186	48.44
	No^a^	34	8.85
	Yes^b^	164	42.71
**Touching dead bat with bare hands is okay**	Don’t know^b^	11	2.86
	No^a^	335	87.24
	Yes^b^	38	9.90
**Bats are important for economic status**	Don’t know^b^	59	15.36
	No^a^	239	62.24
	Yes^b^	86	22.40
**Bats are not harmful animals**	Don’t know^b^	43	11.20
	No^a^	173	45.05
	Yes^b^	168	43.75
**When I touch a bat, can’t get diseases**	Don’t know^b^	55	14.32
	No^a^	68	17.71
	Yes^b^	261	67.97
**People can’t get diseases from bats by sharing drinking water with them**	Don’t know^b^	55	14.32
	No^a^	48	12.50
	Yes^b^	281	73.18
**I feel safe when I enter a place with bats**	Don’t know^b^	6	1.56
	No^a^	320	83.33
	Yes^b^	58	15.10
**It is fine to consume bats**	Don’t know^b^	18	4.69
	No^a^	313	81.51
	Yes^b^	53	13.80
**I am not concerned about diseases that people can get from bats**	Don’t know^b^	50	13.02
	No^a^	159	41.41
	Yes^b^	175	45.57
**I allow my children to touch bats**	Don’t know^b^	9	2.34
	No^a^	322	83.85
	Yes^b^	53	13.80
**When I find a dead bat, I bring it home and cook it**	Don’t know^b^	7	1.82
	No^a^	355	92.45
	Yes^b^	22	5.73
**Perception overall**	Negative	242	63.19
	Positive	141	36.81

^a^Positive perception

^b^Negative perception

### Socio-demographics, knowledge aspects, and perception towards bat exposure among persons living near bat roosting sites in Bundibugyo district

In this study males were more significantly exposed than females (X^2^ = 7.1, *p* = 0.008). Similarly, participants who lived in urban setting compared to rural settings were more exposed (X^2^ = 15.5, *p* =  < 0.001). By occupation, hunters were more exposed than farmers (X^2^ = 13.2, *p* = 0.007). Interestingly, participants with good knowledge about bats had high frequency of exposure compared to those with poor knowledge (X^2^ = 6.6, *p* = 0.007). It was a similar trend for those that had positive perception towards bats compared to those who had negative perception (X^2^ = 21.2, *p* =  < 0.001). Good knowledge on measures put in place to keep bat borne diseases had a high frequency of exposure compared to those with poor knowledge (X^2^ = 15.8, *p* = 0.005). Participants with a positive perception as “bats are important for economic status”, “bat guano is safe to use as fertilizer” and “I feel safe to enter an area that has bats” were more significantly exposed than those with a good perception (X^2^ = 28.5, *p* =  < 0.001), (X^2^ = 63.3, *p* = 0.011) and (X^2^ = 31.9, *p* =  < 0.001) respectively as shown in Table 4.

**Table 4:** Knowledge aspects, perception and risk factors associated with bat exposure among persons living near bat roosting sites in Bundibugyo district

Variable	Exposure		X^2^ (*p*-value)	aOR (95%CI)	*p*-value
	**No (*n* = 170)**	**Yes (*n* = 214)**			
**Sex**					
Female	89(51.7)	83(48.3)		1.0	
Male	81(38.2)	131(61.8)	7.1 (0.008)	1.6(1.0, 2.4)	0.038
**Area setting**					
Rural	131(51.4)	124(48.6)		1.0	
Urban	39(30.2)	90(69.8)	15.5 (< 0.001)	1.9(1.2, 3.1)	0.010
**Occupation**					
Farmer	106(45.9)	125(54.1)		1.0	
Hunter	1(5.0)	19(95.0)	13.2 (0.007)	10.9(1.4, 87.6)	0.024
Others	63(47.4)	70(52.6)	13.2 (0.785)	0.8(0.6, 1.4)	0.551
**Knowledge**					
Poor	107(50.5)	105(49.5)			
Good	63(36.6)	109(63.4)	6.6 (0.007)		
**Perception**					
Negative	129(53.3)	41(29.1)			
Positive	113(46.7)	100(70.9)	21.2 (< 0.001)		
**Bat borne diseases known**					
Good	106(62.4)	64(37.7)		1.0	
Poor	149(69.6)	65(30.4)	6.4 (0.134)	0.6(0.4, 0.9)	0.019
**Measures put in place to keep bats borne diseases**					
Poor	54(31.8)	98(45.8)		1.0	
Good	116(68.2)	116(54.2)	15.8 (0.005)	0.7(0.4, 1.1)	0.112
**Bats are important for economic status**					
Negative	153(90.0)	145(67.8)			
Positive	17(10.0)	69(32.2)	28.5 (< 0.001)		
**Bat guano is safe to use as fertilizer**					
Negative	162(46.3)	188(76.3)		1.0	
Positive	8(23.5)	26(53.7)	63.3 (0.011)	2.5(1.0, 5.9)	0.045
**I feel safe to enter an area that has bats**					
Negative	163(50.0)	163(87.9)			
Positive	7(50.0)	51(12.1)	31.9 (< 0.001)		

### Factors associated with bat exposure among persons living near bat roosting sites in Bundibugyo district

Table 4 shows the final multivariable models describing socio-demographic characteristics, knowledge, and perception risk factors for bat exposure. Socio-demographic characteristics (sex, area setting, and occupation), knowledge (Bat borne diseases known, Measures put in place to keep bats borne diseases) and perception (Bats are important for economic status, Bat guano is safe to use as fertilizer, I feel safe to enter an area that has bats) and their related variables that were significant (*p* < 0.05) were considered for inclusion in the multivariable model, however overall knowledge and overall perception were excluded (model 1). Several variables potentially related with knowledge and perception were considered for inclusion apart from (I feel safe to enter an area that has bats and bats are important for economic status) in the multivariable model (model 2). Sex, area setting, occupation, bat borne diseases known, measures put in place to keep bats borne diseases, and bat guano is safe to use as fertilizer variables were considered for inclusion in the multivariable model. Being male (95% CI: 1.0, 2.4 *p*-value = 0.038) staying in an urban environment area (95% CI: 1.2, 3.1 *p*-value = 0.010), being a hunter (95% CI: 1.4 to 87.6 *p*-value = 0.024), poor knowledge on bat borne diseases (95% CI: 0.4 to 0.9 *p*-value = 0.019), positive perception to bat guano being safe as fertiliser (95% CI: 1.0 to 5.9 *p*-value = 0.045) was associated with a greater odds of exposure. The strongest association was observed with occupation status. After adjusting for other factors, hunters were 10 times more likely than farmers, males were 1.6 times more likely than females, staying in urban were 1.9 times more likely than the rural residents, poor knowledge on bat borne diseases were 0.6 times more likely than good knowledge and positive perception on bat guano being safe as fertilisers were 2.5 times more likely than the negative perception to be exposed to bats. Based on the provided data, the adjusted odds ratio of 0.7 for the good measures (95%CI: 0.4 to 1.1 *p*-value = 0.112), suggests a potential protective effect on the risk of bat exposure.

### Community perceptions of activities associated with bat exposure and risk of bat-borne disease

A total of 7 activities were identified by the 10 groups (Table 5) that were perceived to contribute to bat exposure and bat-borne disease transmission. Hunting during an outbreak (especially of bats), consumption of bats, eating fruits/foods that had been nibbled by bats, presence of bats in a house, and regularly visiting caves or other bat roosts were the most frequently identified. Hunting during an outbreak (especially of bats) was the top ranked activity and perceived as most important in 10 out of 10 groups. Overall, there was a moderate statistical agreement in the ranking of all 7 risk activities across the 10 focus groups (W = 0.52; *P* < 0.01; *n* = 10).

**Table 5:** Community perceptions of activities associated with bat exposure and risk of bat-borne disease transmission in Bundibugyo district, Uganda, 2022. Proportional piling scores for the seven identified risk factors are indicated for each group (*n* = 10). The overall rank was derived from the mean score across all groups

Rank	Activity	FGD1	FGD2	FGD3	FGD4	FGD5	FGD6	FGD7	FGD8	FGD9	FGD10	Mean score
1	Hunting during an outbreak (especially of bats)	29	32	35	24	23	11	24	30	27	36	27.2
2	Consumption of bats	23	26	25	32	21	34	20	24	26	25	25.6
3	Eating bat nibbled fruits/foods		18	18	11	17	10	17	12	10	20	13.3
4	Presence of bats in a house	19	20	16	26		18		10	14	10	13.3
5	Regularly visiting caves or other bat roosts	14		22	7	10	9	21	13		5	10.1
6	Movement of people (immigration, inter-district/inter-community, and emigration)	15	4			29		2		23		7.3
7	Participating in mining						18	16	11		4	4.9

Wa 0.52, *p* < 0.0

^a^Interpretation of Kendall’s coefficient of concordance: W < 0.46, *P* > 0.05 (weak agreement); W = 0.46–0.52, *P* < 0.05 (moderate agreement); W > 0.52, *P* < 0.01 (strong agreement). *FGD* Focus Group Discussion

## Discussion

In general, this study contributes to the understanding of knowledge, perceptions, and exposure to bats in communities living around bat roosts. The findings have implications for viral haemorrhagic fever prevention and control by informing targeted interventions, identifying risk factors, and providing valuable insights into community behaviors and practices. Due to the recent EVD outbreak in Uganda, bat exposure associated risks that result from bat-human interaction needs to be investigated and documented. Although the source of the 2014–2016 Ebola outbreak remains unknown, it may have begun with a single spillover event involving initial bat contact, which underscores the health risks of interacting with bats without appropriate precautions [23]. The history of bat exposure depended on several factors such as bat bites, consumption of bats and having bats within the houses. In this study, 55.7% had a history of bat exposure which could be due to staying near the bat roosting sites such as caves and carrying out fruit farming. We suspect that many of the people staying near bat roosting sites have had interactions with bats for a long time and not necessary at the time of study. However, knowledge of bats was associated with increased acceptance of cohabitation with bats both directly, as well as indirectly, via reduced negative emotions toward bats. Moreover, increased perceived COVID-19 risk was associated with negative emotions toward bats [24]. These results bear resemblance to the Prokop et al. (2009) study, in which a belief in myths about bats was associated with avoidance of bats [24].

Males were 1.6 times more likely to be at risk due to exposure of bats compared to the females. In a study done in Kenya, hostile behavior toward bats (such as hunting the bats) was more common among males which is similar in Bundibugyo district [13]. A study found that females were more likely to report a negative emotions (fear, disgust and anxiety) toward bats and less acceptance of cohabitation with bats [24]. Our results mirror findings on gender differences from previous literature, in which females have shown more negative attitudes toward bats and males being more at risk [25]. Bat hunters have hunted and consumed bats for generations. To them, bat meat is a delicious seasonal food that helps them increase their income. Respondents who carried out hunting were 10 times more likely to have risk factors associated with bat exposure compared to farmers. The occurrence of purposeful human interactions with bats, such as hunting for food (e.g., bushmeat), has been identified in several parts of the world and can pose a risk to human health through spillover of zoonotic pathogens from bats to humans [26]. Bat hunters raise domestic animals at home, where they also butcher and prepare bat meat, sometimes keep bats alive and throw leftover bat body parts or feed it to pets, increasing the risk of disease transmission from bats to animals and humans. A human NiV outbreak in Malaysia occurred when bats infected pigs and pigs infected humans [27].

Among the participants who participated in this study, 54.4% had poor knowledge on factors associated with bat exposure. This indicated that knowledge is associated with bat exposure as awareness of those outbreaks relating bats were unheard by the respondents. A similar study found that residents in Tioman Island had poor knowledge regarding bats, particularly that related to its ability to transmit infections [7]. This study differs from a study done in Nepal which showed that the knowledge regarding Dengue Fever was lower among highland community members, as consequence of lower exposure to the vectors and the diseases, when compared to lowland communities [28]. Knowledge of the disease is considered the first steppingstone to any good measures such as health education activity that is implemented. Knowing the causes and transmission sources of a disease, increases the likelihood that people will become more aware of the spread of bat borne diseases, and of the preventive measures to slow transmission [15].

Positive perception to bat guano being safe as fertiliser had 2.5 higher odds to risk of bat exposure compared to negative perception. The potential risk associated with guano mining is even more stark given that moribund bats (i.e., those most likely to be rabid) normally fall to the floor of caves, where they can readily come in contact with someone collecting bat droppings by hand [29]. The low levels of awareness and understanding about the beneficial role of bats in the ecosystem, coupled with suspected associated risk of bats’ borne zoonoses has led to mass persecution of bats in many parts of world including the Ugandan setting [30]. The effectiveness of any bat-borne diseases prevention strategy may hinge upon how well it diffuses into these communities where guano mining being used on the farms regularly occurs. A study similar to our study found the perceived risk that bats pose to human health was also high, with 93% indicating some degree of risk [30]. In addition, most at risk was hunting during an outbreak (especially of bats) and consumption of bats as these got the highest number during the proportional pilling. The risk varies depending on specific circumstances, so individuals assess their own risk and if deemed higher (e.g. large number of bats in care resulting in a higher rate of interaction), take higher level precautions to prevent transmission [31]. These findings are similar to a study that aimed at understanding under what circumstances the handling bats with unprotected skin occurred i.e., bats trapped on fences, located in residences, hunting etc. bat exposures are most common when humans interacted with trapped or sick bats as they were more likely to carry a lyssavirus [23]. Global pandemics and the linking of diseases to bats can also increase support for bat culling [32]. In many parts of the world, bats have been persecuted as a consequence of their role as the probable origin of SARS-CoV-2 [16, 23].

This study had a few limitations where the proportional piling used only ranking the factors to tally them with the quantitative however, we believe that this was adequate to assess the risk factors for bat exposure and bat borne diseases. Nevertheless, the current study was strengthened by a large sample size for both focus group discussion and survey methods. The study will facilitate a one health approach in dealing with bats and bat borne diseases in Bundibugyo district and having interventions to reduce bat-human interactions. This study serves as a baseline for future investigations of the bat human exposure risk interface and prevalence in Bundibugyo district, and to perform comparative analyses between different regions of the country to understand spatiotemporal variation in bat exposures as they relate to risk of bat borne diseases.

## Conclusions

These findings demonstrate that exposure to bats in communities near bat roosts is common but recognition of the potential practices contributing to spillover of bat-related diseases is still low. Therefore, our results may not be representative of the entire country but provide valuable information for initiating programs to increase awareness among at-risk populations regarding the potential risk of bat exposures, and to communicate the availability of effective PEP in case of wildlife and bat bite exposures. There is a need for educational outreach to raise awareness of bat-associated diseases, prevent exposures to bats through limiting bat-human practices, particularly among communities that practice hunting of wildlife. The data also highlights the need for targeted health communication and education efforts to address these knowledge gaps and promote accurate understanding of bats and disease transmission. Our findings are relevant for the study and risk assessment of other bat pathogens from those that have been exposed to bats within this community.

### Supplementary Information

**Supplementary Material 1.**

## Data Availability

All data supporting the results and conclusion of this paper are included in the article.

## Abbreviations

EVD: Ebola Virus Disease
MVD: Marburg Virus Disease
SARS: Severe Acute Respiratory Syndrome
SEBOV: Sudan Ebola Virus
BEBOV: Bundibugyo Ebola Virus
OR: Odds Ratio
FGD: Focus Group Discussion
