# Supplementary material for: Knowledge, perceptions, and exposure to bats in communities living around bat roosts in Bundibugyo district, Uganda: implications for viral haemorrhagic fever prevention and control
Source: BMC Infect Dis. 2024 Mar 14;24:311. doi: 10.1186/s12879-024-09162-x (PMC10938820; doi:10.1186/s12879-024-09162-x)
Supplement: Supplementary file 1 — Supplementary Material 1. [file 12879_2024_9162_MOESM1_ESM.docx]

**Supplementary documents**

**INFORMED CONSENT FORMS FOR RESEARCH PARTICIPANTS**

**Background and rationale for the study**

Hello Sir/Madam, my name is……………………………………………., and I am part of a PhD student from Makerere University College of veterinary medicine, Biosecurity and Animal resources conducting a study to assess the bat ecology and social epidemiology of filoviridae haemorrhagic fevers in Uganda.

**Purpose of the study:**

Pathogen circulation in reservoir hosts is an essential precursor to spillover, but its ecological aspects are often poorly understood relative to post-spillover processes. Bats are an important clade to study, as they host a uniquely rich set of viruses, including many important emerging zoonotic filoviruses. This study is aimed at elucidating the ecological determinants that favour maintenance/presence of filoviruses at the human-bat interface, and the subsequent processes leading to infection and virus shedding.

**Procedures:**

The study is to be will aim at accelerating the level of introduction of filovirus from feacal matter, bat behaviour and bat distribution, anthropogenic factors and modelling filoviruses distribution to determine the frequency of their introduction into the human population. Cross sectional study design will be used for this research.

**Who will participate in the study and where the study is going to be conducted from?**

The study will be conducted in areas including Bundibugyo, Mubende and Kassanda districts to raise awareness on environmental factors affecting bats ecology, their environmental reservoirs and social epidemiological dynamics by providing scientific evidence for policy and public health strategy development against bat-borne zoonotic illness outbreaks in Uganda.

**Potential risks and discomfort**: There will be minimal risks or discomfort from participating in this study.

**Benefits of the research**: Results from this study will inform about previous spillover events of filoviruses in the Rwenzori and will help to have interventions in place within the community.

**Compensation for participation in the study**: Questionnaires, Key informant guides, field sample collection and in-depth interview guides will be used to collect the data. The interview will take approximately 20 to 30 minutes and participation is voluntary.

**Reimbursement**: research participants who will be told to come to the study site will have to be reimbursed their transport and this will depend on where they have come from with a minimum of shs 10,000 per participant.

**Voluntary participation**: Participation in this study is voluntary and will not affect you. If you don’t wish to participate in this discussion, you can ask to be excused. Even as you answer questions asked to you, you can decide to refuse to answer any question if you do not feel comfortable to do so.

**Confidentiality**: All collected information from this interview will be kept confidential. During the discussions, we shall not use your names but rather assign you with numbers to further protect your privacy. The collected data will also only be accessible to the study investigators and will be kept in a secure place.

**Compensation**: For the time of participation in this study we will provide you with a drink and a snack during the interview process.

**Question**: Do you have any questions about the study that you would like me to attend to?

**Contact information.**

In case you have any other questions or concerns about this study later, you may contact Ms. Ninsiima Lesley Rose the Principal Investigator of the survey at telephone numbers: 0785254323 or 0703186003 and Email: lninsiima04@gmail.com.

If you have any questions about your rights as a participant or you feel that you have been harmed by taking part in the study, please contact Mr. Paul Kutyabami, the Chairperson of the School of Makerere University College of Health Sciences Research and Ethics Committee Email: paulkutyabami@yahoo.com.

Thank you.

**Statement of informed consent**

I would now like to get your consent to participate before I proceed. Do you consent to participate in the study

**Respondent response**: Yes or No

I understand the procedures described above. My questions have been answered to my satisfaction and I agree to participate in the study.

Do you agree with the recording: Yes or No?

Name of the research participant ………………………………………. Age ……………….

Signature /thumb print of participant……………………. Date…………………………

Name and signature/thumbprint of witness………………………………………………………. Date…………………………

Signature of the interviewer………………………………… Date…………………………

**Questionnaires** *assess the knowledge attitude and bat exposure in Bundibugyo district.*

**Section 1: Screening**

| **Questionnaire Code** | **Question** | **Response** |
| --- | --- | --- |
| Q1-01 | Sub-county | 1. Ntandi 2. Burondo 3. Harugale |
| Q1-02 | Area setting | 1. Urban 2. Rural |
| Q1-03 | Household ID | N001  B001  H001 |
| Q1-05 | Date of Interview | ___(dd)/___(mm)/202_ |
| Q1-06 | Respondent has lived in this area > 1 year | 1. Yes 2. No (STOP) |
| Q1-07 | Have you interfaced with bats in any aspects (e.g., clean bat guano from your household)? | 1. Yes 2. No (STOP) |

**Socio-demographic Information**

*Note for research assistants: if there are more than 1 eligible respondent in the household, please select only a person who spends much time at home to be interviewed.*

| **Questionnaire Code** | **Question** | **Response** |
| --- | --- | --- |
| Q2-01 | Respondent sex (observe) | 1. Male 2. Female |
| Q2-02 | What is your main occupation? | 1. No occupation/ unemployed 2. Farmer (rice field or other) 3. Hunter 4. Butcher 5. Housewife 6. Shop/ trade (in market) 7. Government officer or employee of government offices 8. Factory worker 9. Student 10. Restaurant worker 11. Other, specify________________________ |
| Q2-03 | What is your current marital status? | 1. Single 2. Cohabitating/ Married 3. Divorced/ Separated/ Widowed |
| Q2-04 | How many people (including yourself and children) live in this household? | ……………………………………… |
| Q2-05 | How many children (<18 years old) live in this household? | __________________ |
| Q2-06 | What is the highest level of education attained? | 1. Primary 2. Secondary 3. Tertiary 4. None |
| Q2-07 | Could you please estimate your household income per month (Ugx)? | 1. < 15,000 2. 15,001 – 40,000 3. 40,001 – 70,000 4. > 70,001 |

**Knowledge on bats**

| What bat-borne diseases have you heard of? | 1. Ebola 2. Marburg 3. Others specify………………. |
| --- | --- |
| What is the most trusted information regarding to diseases or disease prevention or disease outbreak? (Rank from most to least) | 1. Newspaper 2. Radio 3. Television 4. Social media 5. Family members 6. Health personnel 7. Village health volunteer 8. Neighbor 9. Teacher 10. Priests 11. Nothing 12. Other, please specify________________________ |
| Once you get sick, which person/place do you visit/ talk to first (aside from your family members? | 1. Health personnel in clinics or government health center 2. Go to a drug shop. 3. Medical doctor/ nurse in hospital 4. Traditional healer 5. Village health volunteers 6. Neighbor 7. Teacher 8. Priests 9. No one 10. Other, please specify________________________ |
| What do you think is the source of these frequent outbreaks | Immigration of refugees  Eating other wild game (specify……….)  Eating bats  Trade in wild animals  Climate change (weather)  Other ……………… specify. |
| Have you/family member ever been bitten by a bat? | Yes  No (Skip to Q3.2-04) |
| Have you/family member ever been bitten by any other wild animal | Yes (if yes, specify………………)  No |
| As Q3.2-01, what did you do when you were bitten by the bat? | 1. Do nothing. 2. Clean wound by water immediately 3. Clean wound by water with soap immediately 4. Clean wound by water with soap as well as medicine immediately 5. See doctor/ nurse at health facilities. 6. Use herbal remedies.   Other, please specify ________________________ |

**Practice towards bats among local people**

| **Question** | **Responses** |
| --- | --- |
| Have you or any member of your household ever consumed bats? | 1. Yes 2. No |
| Has/does any of your friends or relatives consume bats | 1. Yes 2. No |
| How did you get the bat? | 1. Catch from caves. 2. Catch from forest, but not in caves. 3. Catch within community/village. 4. Pick up from carcass in household or in community. 5. From hunter 6. Bought from vender in the market. 7. From relatives (e.g., parents) 8. From neighbor 9. Other, please specify ___________________________ |
| If bought, where was it sold? | 1. Local market within the area 2. None, it was sharing within family members. 3. Hunter 4. Neighbor 5. Other, please specify _________________________ |
| Did you slaughter it yourself? | 1. Yes 2. No |
| After cooking does every member of the household take part of eating the meal (multiple choice) | 1. Wife 2. Husband 3. Parents 4. Children (age <10 years old) 5. Neighbor 6. Vendor 7. Hunter   Other, specify _____________________________ |

**Perceptions towards bats to local people**

***Instruction for asking questions of this section***

*Now, I’m going to read you some statements about bats. Please tell me if you feel –*

1. *Yes*
2. *No*
3. *Don’t know*

| **Questionnaire Code** | **Question** | **Interviewer fills the answer (as number)** |
| --- | --- | --- |
| Q4-01 | People can get diseases from bats |  |
| Q4-02 | I’m not concerned about disease that people can get from bats |  |
| Q4-03 | Bats are not harmful animals. |  |
| Q4-04 | Bats are important for economic status in this community |  |
| Q4-05 | I can get diseases from bats if I touch them |  |
| Q4-06 | People can get diseases from bats by sharing drinking water with them |  |
| Q4-07 | People can’t get diseases from bat by eating fruits that might be bitten by bats |  |
| Q4-08 | Bat guano is safe to use as fertilizer |  |
| Q4-09 | It is fine to consume bats |  |
| Q4-11 | I allow my child/ren to touch bats |  |
| Q4-12 | When I find a dead bat, I will bring it home and cook as sauce. |  |
| Q4-13 | I feel safe to enter an area that has bats. |  |
|  | Touching dead bat with bare hands is okay |  |

**Behaviour of individuals towards bats**

*Instruction for asking questions of this section*

*We are going to discuss on the bats in several aspects. Please feel free to share the information. There is no right or wrong answer. We would like to learn from you to understand your experience regarding your interaction with bats in your community.*

| **Question** | **Response** |
| --- | --- |
| Where are the bats usually found | In houses through broken ceilings  In bat roosts on farms  In caves  At workplaces  In school buildings  In other areas (Please specify……...) |
| Why do you go where these bats live? | For religious activities  For recreation  To collect bat guano  To fetch water  To hunt for bats  To farm  For other reasons |
| Do you have bats in your household | 1. Yes  2. No |
| If Q3.1-01 answer No, have you been in a household with bats? | 1. Yes  2. No |
| Did you or anyone in your household find a dead bat in your household? | 1. Yes 2. No (Skip to Q3.1-03) |
| As Q3.1-01, what did you/ your household members do? | 1. Did nothing 2. Cooked it as sauce. 3. Threw it to waste pit in the community/ household. 4. Burn it. 5. Bury it. 6. Other, please specify ________________________ |
| Have you/your household members ever found feces of bats in your house? | 1. Yes 2. No (Skip to Q3.2-07) |
| If Q3.1-04 answer Yes, where did you find the fecal matter? | 1. food 2. water 3. Utensils 4. Clothes 5. Others…………. specify |
| Have you/your household members ever found dead bats in your community (but it is not in household e.g., community’s park, or rice field)? | 1. Yes 2. No (Skip to Section 3.2) |

**THANK YOU FOR YOUR PARTICIPANT**
